# Predicting Phenotypic Severity of Uncertain Gene Variants in the *RET* Proto-Oncogene

**DOI:** 10.1371/journal.pone.0018380

**Published:** 2011-03-30

**Authors:** David K. Crockett, Stephen R. Piccolo, Perry G. Ridge, Rebecca L. Margraf, Elaine Lyon, Marc S. Williams, Joyce A. Mitchell

**Affiliations:** 1 Biomedical Informatics, University of Utah School of Medicine, Salt Lake City, Utah, United States of America; 2 ARUP Institute for Clinical and Experimental Pathology, Salt Lake City, Utah, United States of America; 3 Intermountain Healthcare Clinical Genetics Institute, Salt Lake City, Utah, United States of America; Krebsforschungszentrum, Deutsches, Germany

## Abstract

Although reported gene variants in the *RET* oncogene have been directly associated with multiple endocrine neoplasia type 2 and hereditary medullary thyroid carcinoma, other mutations are classified as variants of uncertain significance (VUS) until the associated clinical phenotype is made clear. Currently, some 46 non-synonymous VUS entries exist in curated archives. In the absence of a gold standard method for predicting phenotype outcomes, this follow up study applies feature selected amino acid physical and chemical properties feeding a Bayes classifier to predict disease association of uncertain gene variants into categories of benign and pathogenic. Algorithm performance and VUS predictions were compared to established phylogenetic based mutation prediction algorithms. Curated outcomes and unpublished *RET* gene variants with known disease association were used to benchmark predictor performance. Reliable classification of *RET* uncertain gene variants will augment current clinical information of *RET* mutations and assist in improving prediction algorithms as knowledge increases.

## Introduction

Medical genetics involves diagnosis, management, and determining risk of hereditary disorders [1], [2]. The genotype:phenotype correlation of gene variants in disease is a major component of medical genetics. In monogenic diseases, gene mutations are typically curated as either pathogenic or benign. However, many gene variants must be classified as “unknown” or “uncertain” significance because they have not been clearly associated with a clinical phenotype.

The outlay of time and labor to validate the disease association concerning a variant of uncertain significance (VUS) within the coding portion of a gene can be daunting and cost prohibitive [3], [4]. This is in large part, due to the communication between clinicians and laboratory geneticists needed to resolve these variants [5], [6]. To help bridge this genotype:phenotype gap, the use of machine learning classification algorithms to narrow the uncertain “grey area” between pathogenic and benign sequence variants warrants careful evaluation [7], [8], [9], [10]. Reliable machine learning based classification may augment costly patient recruitment, family histories, and biochemical confirmation of a gene variant with no associated disease correlation [11], [12], [13].

There are established methods for predicting mutation severity based on amino acid substitution penalties, structural disruption, sequence homology (ortholog conservation) or neural nets, such as PolyPhen [13], SIFT [14], MutPred [9] and PMut [15]. However, prediction algorithms are not always in agreement with curated data or each other [16], [17], [18]. Thus, there are opportunities to explore the use of other informatics approaches to this problem. Machine learning methods that can be trained on data available in well-curated gene variant collections may be promising tools to improve the predictive capabilities available to the research community.

**Figure 1:**
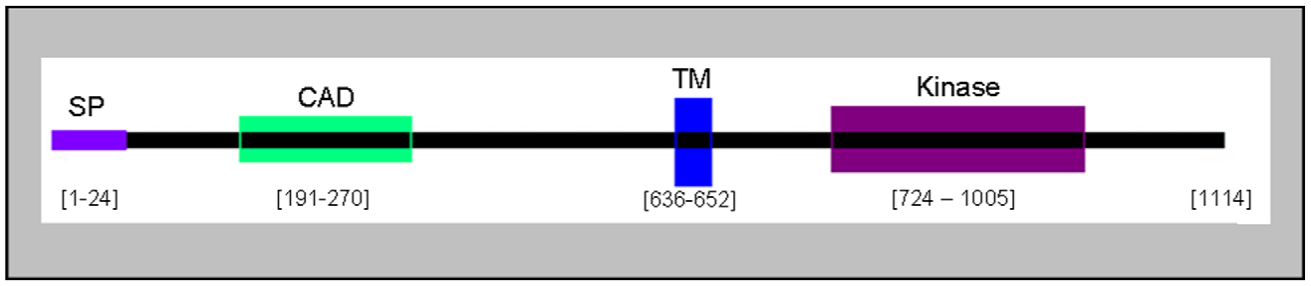
Schematic of the full length 1114 amino acid RET protein showing the signal peptide (SP, residues 1-24), cadherin domain (CAD, residues 191-270), transmembrane domain (TM, residues 636-652), and tyrosine kinase motif (Kinase, residues 724-1005).

The human *RET* gene (REarranged during Transfection) is located on chromosome 10q.11 codes for 20 exons. The transcript length is 5,659 bps and translates to the 1,114 amino acid residue protein (UniProt RET_HUMAN, #P07949) as shown in Figure 1. The gene belongs to the cadherin superfamily and encodes a receptor tyrosine kinase which functions in signaling pathways for cell growth and differentiation. *RET* plays a critical role in neural crest development. It can also undergo oncogenic activation *in vivo* and *in vitro* by cytogenetic rearrangement. It can be further classified by Gene Ontology (GO) categories (www.geneontology.org) of biological process of homophilic cell adhesion, posterior midgut development, and protein amino acid phosphorylation. Its GO annotated cellular location is component integral to membrane and the GO category of molecular functions lists ATP binding, calcium ion binding and transmembrane receptor protein tyrosine kinase activity. Functional domains of the RET protein are also summarized in Figure 1.

*RET* is essential for the development of the sympathetic, parasympathetic and enteric nervous systems. Disruption of function by germline mutations in *RET* have been associated with several diseases in humans including three related inherited cancers: multiple endocrine neoplasia type IIA (MEN2A), multiple endocrine neoplasia type IIB (MEN2B), and familial medullary thyroid carcinoma (FMTC). [19], [20] RET has also been implicated in congenital aganglionosis (absence of enteric nerve cells) in the gastrointestinal tract (Hirschsprung's disease) lack of the neuroenteric plexi impairs smooth muscle activity of the intestines (particularly the colon) resulting in refractory constipation. [21]

Although well understood codon changes often guide patient therapy or surgical options [22], *RET* gene variants may vary in functional severity, where some are reported as benign, some pathogenic, and some of uncertain significance. Curated *RET* oncogene mutations have been recently reported by Margraf et al. [23] The disease classification of *RET* gene variants has been curated as benign (6%), pathogenic (52%) and VUS (42%), meaning unknown or uncertain association with disease or phenotype outcome. This archive currently hosts 146 *RET* variants, including 62 VUS entries that can be accessed at http://www.arup.utah.edu/database/.

Accurate prediction of disease association for novel mutations and uncertain gene variants is of great importance to medicine and biology. Informatics tools for predicting disease severity of uncertain gene variants will aid in the improvement of genetically-informed patient care. With a rapidly growing number of on-line resources for gene variants collections, the opportunity to apply machine learning algorithms to well curated disease causing gene sets becomes increasingly desirable.

The absence of any gold standard for predicting phenotype severity in uncertain gene variants prompts two questions. Are algorithms trained specific to a gene/disease setting more appropriate to use than generalized on-line prediction tools? Does agreement between several and varying algorithms influence clinician decision-making? This study expands a recently reported algorithm, we here term Primary Sequence Amino Acid Properties (PSAAP), which uses feature selected amino acid physicochemical properties of primary amino acid sequence [24]. This previous work detailed algorithm performance using only gene variants with known disease association, while here we report applying the PSAAP algorithm classification for pathogenicity of novel and uncertain gene variants found in the *RET* proto-oncogene into categories of benign or pathogenic. The PSAAP algorithm performance has also been compared to four well-established prediction tools available on-line and agreement between algorithms summarized.

## Results

The independent test set of *RET* curated mutations was used to evaluate performance of different categories of classifier algorithms. The best performing algorithm (using Weka) was Naïve Bayes. Algorithm metrics for this novel Bayes classifier of *RET* disease outcome were calculated using the above test set data. Evaluation of the classifier yielded a sensitivity of 0.938, specificity of 0.867 and positive predictive value (precision) of 0.883. Performance for our Primary Sequence Amino Acid Properties (PSAAP) classifier is summarized in Table 1. A benchmark of prediction performance for the established algorithms (MutPred, PolyPhen, PMut and SIFT) was also performed using curated *RET* gene variants with known disease outcomes. Following the 88% of the PSAAP classifier, MutPred was next closest to predicting the correct disease outcomes for the known *RET* variants with 84% precision. PolyPhen yielded the highest specificity for *RET* variant disease association of 92%, yet had the lowest precision at 54%. PMut correctly predicted gene variant disease outcomes with 72% precision but had the lowest specificity at 59%. Table 1 also summarizes performance metrics (sensitivity, specificity, precision) for curated *RET* mutations using the four established prediction algorithms.

**Table 1:** PSAAP algorithm performance of predicted phenotypes using curated *RET* mutations.

	**PSAAP Prediction** a	**MutPred Prediction^b^**	**PolyPhen Prediction^c^**	**PMut Prediction^d^**	**SIFT** **Prediction^e^**
**Sensitivity**	0.938	0.767	0.597	0.783	0.816
**Specificity**	0.867	0.823	0.920	0.591	0.821
**Precision**	0.883	0.843	0.541	0.723	0.779

a Primary Sequence Amino Acid Properties (PSAAP) algorithm.

b Analyzed with default settings at http://mutdb.org/mutpred.

c Analyzed with default settings at http://genetics.bwh.harvard.edu/pph.

d Analyzed with default settings at http://mmb.pcb.ub.es/PMut.

e Analyzed with default settings at http://sift.jcvi.org.

Next, evaluation of *RET* non-synonymous VUS mutations (n = 46) was performed using our recently reported algorithm [24]. The PSAAP algorithm classified 22 of the uncertain variants as pathogenic, while the remaining 24 fell within the benign grouping. For those variants classified as predicted pathogenic, the PSAAP algorithm estimated confidence remained above 90%. The classifier predicted disease outcome using our algorithm is listed in Table 2.

**Table 2:** Algorithm agreement for *RET* uncertain gene variants and predicted pathogenicity.

*RET* uncertain gene variant	PSAAP Prediction^a^	MutPred Prediction^b^	PolyPhe Prediction^c^	SIFT Prediction^d^	PMut Prediction^e^
*5/5 agreement*					
A510VR600QK603QE632KA640GV648IY791NE843DR844LR844WR886WR912Q	BenignBenignBenignBenignBenignBenign**pathogenic**benign**pathogenic** **pathogenic** **pathogenic** **pathogenic**	BenignBenignBenignBenignBenignBenign**disrupted**Benign**disrupted** **disrupted** **disrupted** **disrupted**	BenignBenignBenignBenignBenignBenign**probably damaging**benign**probably damaging** **probably damaging** **probably damaging** **probably damaging**	Toleratedtoleratedtoleratedtoleratedtoleratedtolerated**affects function**tolerated**affects function** **affects function** **affects function** **affects function**	Neutralneutralneutralneutralneutralneutral**pathological**neutral**pathological** **pathological** **pathological** **pathological**
*4/5 agreement*					
C611SD631GE805KS819IR833CS904CS904F	**pathogenic** **pathogenic**benign**pathogenic** **pathogenic** **pathogenic** **pathogenic**	**disrupted**benign**disrupted** **disrupted**benignbenignbenign	**Probably damaging** **probably damaging** **probably damaging** **probably damaging** **probably damaging** **probably damaging** **probably damaging**	**Affects function** **affects function** **affects function** **affects function** **affects function** **affects function** **affects function**	neutral**pathological** **pathological**neutral**pathological** **pathological** **pathological**
*3/5 agreement*					
Y606CC531RG533SD631AD631VR635GP841LL881VK907M	**pathogenic** **pathogenic** **pathogenic** **pathogenic** **pathogenic** **pathogenic** **pathogenic**benign**pathogenic**	benignbenignbenignbenignbenignbenignbenign**disrupted**benign	**probably damaging** **probablydamaging** **probably damaging** **probably damaging** **probably damaging** **probably damaging** **probably damaging** **probably damaging** **probably damaging**	toleratedtolerated**affects function** **affects function** **affects function**toleratedtolerated**affects function** **affects function**	**pathological** **pathological**neutralneutralneutral **pathological** **pathological**neutralneutral
*2/5 agreement*					
C630SD631ES649LH665QR844QM848TI852MK907E	**pathogenic**benign**pathogenic**benignbenignbenignbenignbenign	benignbenignbenignbenignbenignbenignbenignbenign	**probably damaging** **probably damaging** **probably damaging** **probably damaging** **probably damaging** **probably damaging** **probably damaging** **probably damaging**	toleratedtoleratedtoleratedtoleratedtoleratedtolerated**affects function** **affects function**	Neutral**pathological**neutral**pathological** **pathological** **pathological**neutralneutral
*1/5 agreement*					
G321RE511KD631NA641SK666NR770QN777SV778IE818K	benignbenignbenignbenignbenignbenignbenignbenignbenign	benignbenignbenignbenignbenignbenignbenignbenignbenign	benignbenignbenign**possibly damaging** **probably damaging** **probably damaging** **possibly damaging**benign**possibly damaging**	toleratedtoleratedtoleratedtoleratedtoleratedtoleratedtolerated**affects function**tolerated	**pathological** **pathological** **pathological**neutralneutralneutralneutralneutralneutral

a Primary Sequence Amino Acid Properties (PSAAP) algorithm.

b Analyzed with default settings at http://mutdb.org/mutpred.

c Analyzed with default settings at http://genetics.bwh.harvard.edu/pph.

d Analyzed with default settings at http://sift.jcvi.org.

e Analyzed with default settings at http://mmb.pcb.ub.es/PMut.

Results from analysis of the *RET* uncertain gene variants (VUS) using the established on-line prediction tools are also summarized in Table 2, with predicted pathogenic variants bolded and ranked by agreement. The MutPred tool calculates the probability of a deleterious mutation and corresponding hypothesis of disrupted molecular mechanism. We used MutPred's default probability cutoff of 0.75 for differentiating between benign and disrupted/pathogenic mutations. Our PSAAP algorithm agreed with MutPred in 16 benign and 8 pathogenic predictions for 52% agreement (24 out of 46). PolyPhen has outcomes of “benign”, “possibly damaging” and “probably damaging”. The PSAAP classifier agreed with PolyPhen in 13 benign and 22 pathogenic predictions for 76% agreement (35 out of 46). PMut yields outcomes of “pathological” or “neutral” and a corresponding reliability metric (lower is better). Our PSAAP trained algorithm was in concordance with PMut in 13 benign and 14 pathogenic predictions for 58% agreement (27 out of 46). The SIFT algorithm gives outcomes of “tolerated” and “affects protein function”. Our algorithm agreed with SIFT in 19 benign and 16 pathogenic predictions for 76% agreement (35 out of 46).

**Figure 2:**
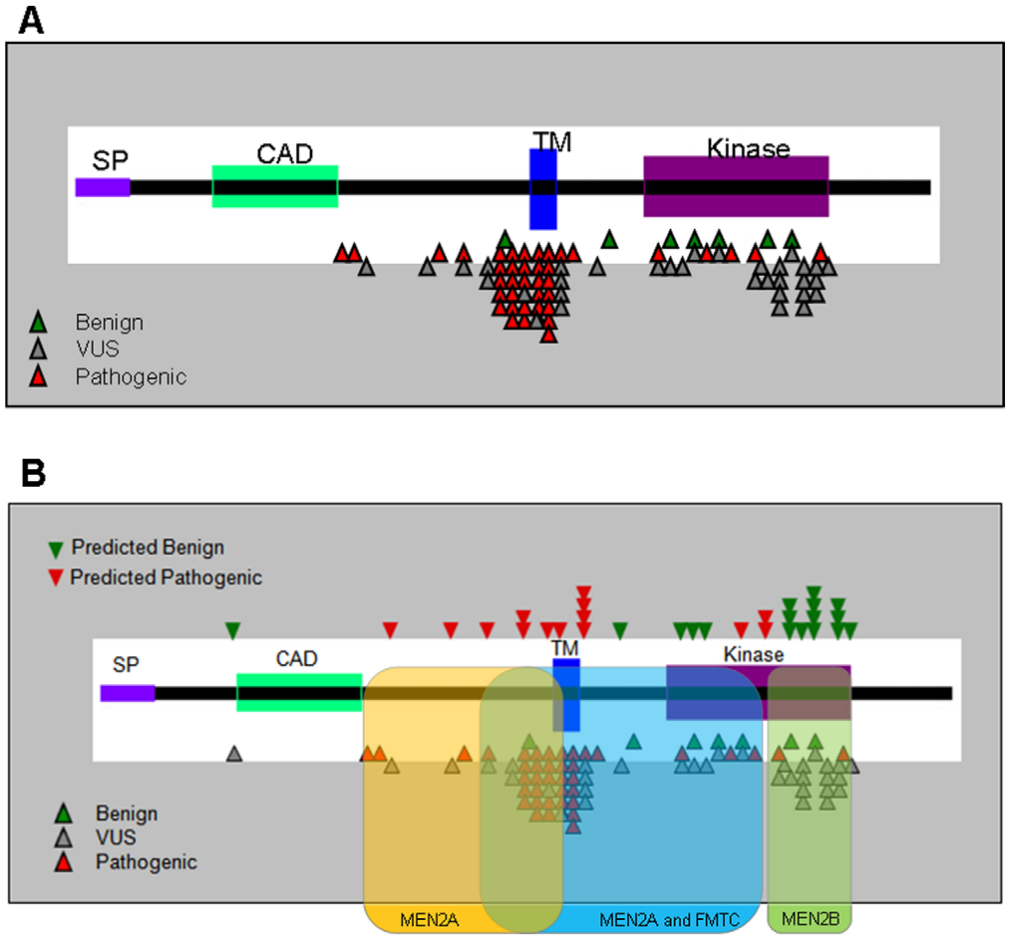
Schematic of the RET protein with A) clinically curated variants and B) predicted disease association for uncertain variants mapped across protein location. The phenotype overlay shows regions of reported MEN2A, MEN2B and FMTC disease.

Of special interest, for predicted *RET* benign variants, 7 of 24 agreed across all algorithms, while only 6 of 22 predicted pathogenic *RET* variants showed agreement across the different methods. Although only 13 out of 46 (28%) were concordant, these variants may count as having a higher degree of confidence in prediction due to the varied methodologies and basis of classification. Importantly, the focus of molecular research and clinical efforts could therefore be directed to this prioritized listing of *RET* uncertain variants. Curated variants are shown mapped across the length of the protein in Figure 2A. This graphing visually highlights the cysteine rich region just prior to the transmembrane domain, and the transmembrane domain itself which contain the majority of pathogenic variants. Our predictions for the uncertain RET variants (VUS) are also mapped by location across the length of the protein as added into Figure 2B.

Finally, several unpublished *RET* gene variants with known pathological (MEN2) outcomes (n = 5) were identified during routine genetic testing at ARUP Laboratories. To further benchmark a gold standard of truth for *RET* mutation prediction, all five algorithms were used to classify this set of not yet seen variants. Our novel Bayes trained PSAAP classifier correctly identified all five variants as pathogenic. PMut called 3 disease causing variants correctly, but classified two others as “neutral” mutations, when in fact these changes were known to be associated with disease. PolyPhen also correctly identified 3 as probably damaging (pathogenic), but missed classified the same 2 variants as PMut. SIFT predicted 4 of these variants would affect function (pathogenic), but called one of the same variants “tolerated.” MutPred correctly predicted all 5 as pathogenic.

## Discussion

Mutations in the *RET* proto-oncogene have been directly associated with MEN2 and hereditary medullary thyroid carcinoma, and provide guidance for patient care. Accurate classification of phenotype severity for novel mutations and uncertain variants as relating to disease is of great importance to proper patient care. Although correlation of genotype-phenotype offers therapy options that would otherwise remain hidden and may lead to disease specific mutation-guided management strategies, appropriate caution is justified when clinicians are asked to trust computational outcomes for determining patient care [6].

On-line mutation prediction tools have been available for many years. Prediction tools such as PolyPhen [13] and SIFT [14] are primarily based on multiple alignment and amino acid substitution penalties. More recently, MutPred [9] which calculates probability of deleterious mutations by disrupted molecular mechanism. Additionally, PMut [15] is neural net based and trained on human mutations. We recently reported classification of curated *RET* gene variants using primary amino acid sequence properties and Naïve Bayes [24]. A key feature to highlight is the fact that the PSAAP algorithm relies on Bayes probability trained on gene-specific and clinically curated disease outcomes. Comparison of this recent PSAAP algorithm with established on-line prediction tools may improve our understanding of predicting mutation status in the *RET* proto-oncogene.

Sorting Intolerant From Tolerant (SIFT) was first published in 2003 by Ng and Heinikoff from work done at the Fred Hutchinson Cancer Research Center in Seattle [14]. The algorithm predicts whether an amino acid substitution will affect the function of a protein based on both sequence homology to various orthologs and physical properties of amino acids. SIFT is a multistep procedure that (1) searches for and chooses similar sequences (2), makes an alignment of these sequences, and (3) calculates scores based on the amino acids appearing at each position in the alignment. It was initially developed and trained on nsSNP data sets from LacI, Lysozyme, and HIV protease [25]. This algorithm works especially well when adequate numbers of sequence homologs are available for multiple alignment. Conversely, poor performance is seen when multiple alignment in not reliable or completely unavailable.

Polymorphism Phenotyping (PolyPhen) is an EMBL based tool from 2002 from Ramensky et al. [13] It was developed to predict the possible impact of an amino acid substitution on the structure and function of a human protein using physical and comparative considerations. It was originally developed from a set of disease-causing mutations in human proteins with known structures extracted from the SWISS-PROT database, and correlated to the Online Mendelian Inheritance in Man (OMIM) database [26]. Since the algorithm relies on predicted structural disruption, it works especially well where protein structure is known and less reliable when a solved protein structure is not available.

MutPred is a recently developed prediction algorithm by Li, Mooney and Radivojac [9]. It builds on the established SIFT method but offers improved classification accuracy based upon protein sequence, and models changes of structural features and functional sites between wild-type and mutant sequences with output of probabilities of gain or loss of structure and function. It was trained on a set of disease SNPs from cancer and the OMIM disease archive. This predicted disruption of molecular function again work especially well for well studied proteins, where homolog and solved structure is available.

PMut was first published in 2005 by the Molecular Modeling Unit at the Institut de Recerca Biomédica, Parc Científic de Barcelona, Spain [15]. It is based on a two layer neural network and was trained using human mutational data. It allows for either prediction of single point amino acidic mutations or scanning of mutational hot spots. Results are obtained by alanine scanning, identifying massive mutations and genetically accessible mutations. A graphical interface for Protein Data Bank (PDB) structures, when available, and a database containing hot spot profiles for all non-redundant PDB structures are also accessible from the PMut server.

**Figure 3:**
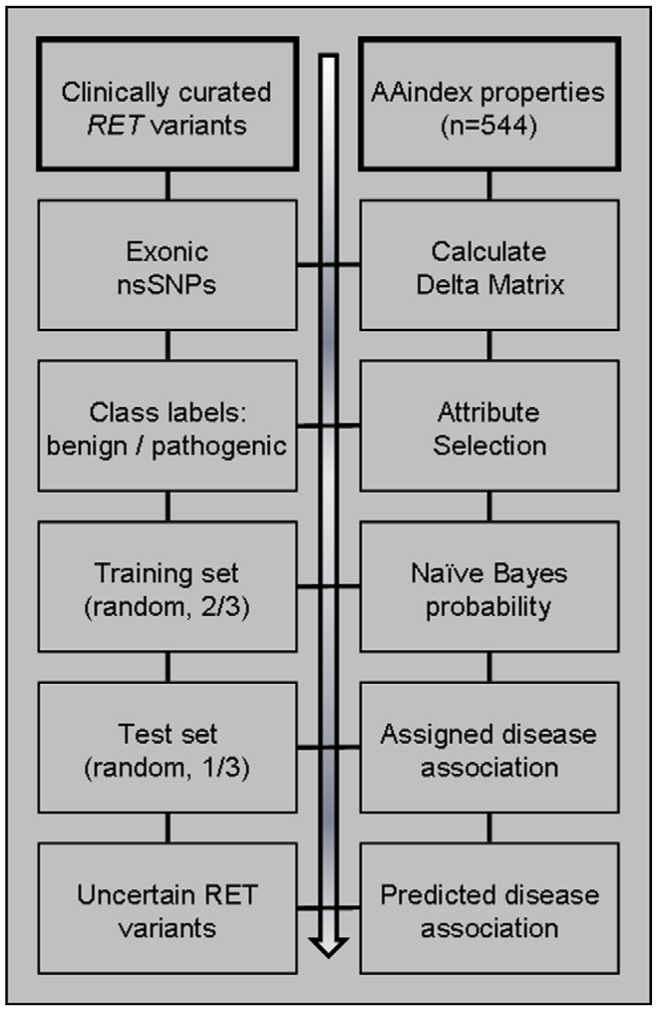
Overview of the PSAAP classifier workflow, highlighting the gene-specific algorithm training on clinically curated disease association.

Benchmarking the established prediction algorithms with curated *RET* variants and associated MEN2 disease demonstrates our PSAAP classifier model compares very well to other established prediction tools. A distinguishing feature of the PSAAP model herein reported is the algorithm was trained specifically to curated *RET* disease outcomes, as summarized in Figure 3 . This is in contrast to the less robust curated collections of mutations such as OMIM or dbSNP. Further, no homolog alignment or solved protein structure is necessary. Rather, it relies on primary sequence information only - with calculated delta matrices of substituted amino acid properties , and is therefore not limited to scenarios where SIFT or PolyPhen (and others) have traditional been used. These facts may explain the improved performance when classifying *RET* variants as compared to generalized prediction tools available on-line.

Ranking agreement of predicted phenotype severity across several complimentary algorithms may provide an additional level of clinical confidence in computational classifiers. At a minimum, these five all-in-agreement “predicted pathogenic” *RET* variants warrant closer investigation by traditional and molecular techniques. Furthermore, algorithm agreement in a clinical setting may be just as important for “benign” as it might be for “pathogenic.”

Personalized treatment in genomic medicine cannot advance until questions such as *what was found*, *what does it mean* and *what to do about it* can be answered for each individual patient and genetic test result. Among the key features critical for a decision support framework in clinical genetic testing is a reliable phenotype classification tool and scoring metric to predict consequences of a variation that alters protein structure. For these uncertain gene variants, the in-house algorithm trained specifically on available *RET* curated outcomes seems to outperform well-established and generalized prediction tools available on-line. More importantly, agreement between several predictors may provide research priority for novel and uncertain gene variants.

The use of machine learning algorithms to classify uncertain gene variants in disease is a promising tool to strengthen our underlying knowledge of disease pathogenesis. Software algorithms to better classify gene variants of uncertain significance are necessary to move translational research forward. This follow up study used the PSAAP algorithm to “reclassify” 46 variants of uncertain significance within the *RET* proto-oncogene into categories of benign or pathogenic. This novel application of classification algorithms for computational prediction of phenotype severity in uncertain gene variants could be generally applied to any gene-disease setting where a corpus of curated gene variants are trusted and where reported mutations impact clinical care.

## Methods

Non-synonymous *RET* variants were characterized by physicochemical differences in primary amino acid sequence resulting from the mutation. Attributes of mutation status were characterized using values of 544 physical, chemical, conformational, or energetic properties (AAindex v9.4) [27]. AAindex is a database of numerical indices representing various physicochemical and biochemical properties of amino acids and pairs of amino acids. For each *RET* variant, matrices of delta values for each biochemical property of the substituted amino acid were calculated by Python scripting and the resulting mutation described by an array of variables archived using SQL - where each matrix corresponds to the absolute value of the difference between the value of the property in the amino acid present in the wild type and the one in the mutant.

As previously described, representative algorithms from different categories of classification (such as nearest neighbor, bayes, regression, rule-based and support vector machine) were evaluated for their ability to correctly predict mutation status in the training set [24]. Briefly, a clinically curated set (n = 84) of non-synonymous *RET* mutations with known pathogenicity was used to train and test machine learning classification algorithms. Although training and test sets included different disease subtypes such as MEN2A (n = 40), MEN2B (n = 3), FMTC (n = 5), MEN2A and FMTC (n = 36) - class labels of “pathogenic” and “benign” were used to describe all curated disease association. Random selection was used to build a 2/3 training set (n = 56) and 1/3 test set (n = 28). Attribute selection (feature selection) was performed during classification training/testing. Machine classification algorithms were implemented using the Weka software package (v3.6) [28]. When a given classification algorithm produced posterior probabilities of mutation status, we assigned each variant's mutation status according to the higher posterior probability (Weka's default behavior).

The PSAAP algorithm performance was evaluated using the test set, with sensitivity (true positive rate), specificity (true negative rate), and positive predictive value (precision) calculated. A data set of non-synonymous *RET* uncertain variants (n = 46) was then analyzed using our PSAAP (Naïve Bayes, gene-specific trained) classification algorithm. The workflow of our PSAAP algorithm is summarized in Figure 3.

Next, both curated *RET* mutations (known disease association) and *RET* uncertain variants (VUS data) were analyzed and compared using four existing mutation prediction algorithms. These established prediction tools are mainly based on phylogenetic properties such as sequence homology, amino acid substitution penalties or structural disruption. MutPred (mutdb.org/mutpred) [9], PolyPhen (genetics.bwh.harvard.edu/pph) [13], SIFT (sift.jcvi.org) [14], and PMut (mmb2.pcb.ub.es:8080/PMut) [15] were accessed during July/August 2010. Both curated *RET* variants and *RET* VUS entries were evaluated using respective default settings.

Finally, several unpublished *RET* disease variants (n = 5) with known pathogenic outcomes (by surgical pathology, molecular testing and family history) were identified during routine genetic testing at ARUP Laboratories. This nascent set of *RET* variants was also analyzed and compared by all prediction algorithms to further benchmark some standard of performance and precision. Data and methods used for this study were approved by the Institutional Review Board of the University of Utah.
